# Mapping the clinical correlates of brain hypoperfusion in behavioral variant frontotemporal dementia: insights from a SPECT imaging study

**DOI:** 10.3389/fnins.2026.1737054

**Published:** 2026-02-04

**Authors:** Electra Chatzidimitriou, Chrissa Sioka, Eleni Aretouli, Ioannis Iakovou, Panagiotis Ioannidis, Despina Moraitou

**Affiliations:** 1 Department of Cognition, Brain and Behavior, School of Psychology, Faculty of Philosophy, Aristotle University of Thessaloniki (AUTh), Thessaloniki, Greece; 2 2nd Department of Neurology, AHEPA University Hospital, Aristotle University of Thessaloniki (AUTh), Thessaloniki, Greece; 3 Laboratory of Neurodegenerative Diseases, Center for Interdisciplinary Research and Innovation, Aristotle University of Thessaloniki (CIRI-AUTh), Thessaloniki, Greece; 4 Department of Nuclear Medicine, University Hospital of Ioannina, Ioannina, Greece; 5 Department of Psychology, School of Social Sciences, University of Ioannina, Ioannina, Greece; 6 2nd Academic Department of Nuclear Medicine, AHEPA University Hospital, Aristotle University of Thessaloniki (AUTh), Thessaloniki, Greece

**Keywords:** behavioral disturbances, Brodmann area, bvFTD, clinical correlates, cognitive deficits, personality changes, rCBF, SPECT imaging

## Abstract

**Background/objectives:**

Behavioral variant frontotemporal dementia (bvFTD) is characterized by heterogeneous cognitive, behavioral, and personality changes, reflecting underlying disruptions in brain structure and function. Neuroimaging, particularly single-photon emission computed tomography (SPECT) assessment of regional cerebral blood flow (rCBF), provides a valuable functional window into these alterations. Despite advances in mapping perfusion abnormalities in bvFTD, the precise relationship between region-specific brain hypoperfusion and the full spectrum of clinical manifestations remains incompletely understood. The present study aims to systematically explore these associations to clarify the neurofunctional underpinnings of bvFTD and to delineate brain-behavior relationships across cognitive, behavioral, and personality domains.

**Methods:**

rCBF was assessed in 25 individuals with early-stage bvFTD using ^99m^Tc-HMPAO SPECT imaging, with analysis of lobar and Brodmann area (BA) perfusion performed via NeuroGam™ software. Participants underwent a comprehensive neuropsychological evaluation to assess cognitive functions, while behavioral disturbances and personality traits were evaluated through informant-based measures. Relationships between rCBF and clinical variables were examined using non-parametric correlation analyses, with correction for multiple comparisons.

**Results:**

Reduced perfusion in right prefrontal regions correlated with heightened behavioral disturbances (BAs 8 and 46) and overall disease severity (BA 8). In addition, greater hypoperfusion in frontotemporal and limbic areas was linked to more pronounced declines in personality traits, including conscientiousness (left BAs 6, 31) and extraversion (right BAs 8, 32; left BA 37). A pattern of statistically significant associations also emerged between rCBF in frontal, temporal, limbic, and parietal regions and domain-specific cognitive performances, including visuoconstructional abilities (right BA 5), memory skills (left BAs 28 and 31), executive functions (left BAs 6, 31; right BAs 5, 8), and social cognition (left BA 37; right BAs 11, 32, 40, 46). In contrast, global cognitive measures showed no significant association with regional perfusion.

**Discussion/conclusions:**

These findings reveal that distinct patterns of regional hypoperfusion are selectively linked to specific cognitive, behavioral, and personality alterations in early-stage bvFTD, highlighting the neural substrates underlying its core clinical features. Identifying discrete brain regions associated with key symptoms may inform the development of more targeted therapeutic interventions, ultimately enhancing personalized management and care for individuals affected by this complex and highly disabling syndrome.

## Introduction

### Behavioral variant frontotemporal dementia

Behavioral variant frontotemporal dementia (bvFTD) is the most common clinical presentation within the frontotemporal lobar degeneration (FTLD) spectrum and a leading cause of young-onset dementia, typically manifesting before 65 years of age (Onyike and Diehl-Schmid, 2013; Bang et al., 2015). This progressive neurodegenerative syndrome is characterized by early and prominent alterations in behavior, personality, social comportment, and emotional regulation, often preceding significant cognitive decline (Rascovsky et al., 2011; Piguet et al., 2011). According to the revised consensus criteria of the International Behavioral Variant FTD Criteria Consortium (FTDC), a diagnosis of possible bvFTD requires progressive behavioral and/or cognitive deterioration and the presence of at least three core clinical features such as disinhibition, apathy, loss of empathy, compulsive behaviors, dietary changes, and executive dysfunction, with relative sparing of memory and visuospatial abilities (Rascovsky et al., 2011). Probable bvFTD additionally requires evidence of significant functional decline and supportive neuroimaging findings, including frontal and/or anterior temporal atrophy on Magnetic Resonance Imaging (MRI) or Computed Tomography (CT) or hypoperfusion/hypometabolism on Single-photon Emission Computed Tomography (SPECT) or Positron Emission Tomography (PET) (Rascovsky et al., 2011). Despite these well-defined diagnostic criteria, the clinical phenotype of bvFTD remains highly heterogeneous, reflecting substantial underlying neuropathological variability that complicates early diagnosis, prognostic accuracy, and therapeutic development (Rascovsky et al., 2011; Piguet et al., 2011; Seeley, 2019).

### Brain perfusion abnormalities in bvFTD

Given the clinical heterogeneity of bvFTD, neuroimaging provides essential diagnostic support by capturing structural and functional brain changes. Structural imaging, such as MRI or CT, identifies regional atrophy, whereas functional imaging evaluates neuronal activity, commonly through measures of cerebral blood flow or metabolism (Tartaglia et al., 2011). Regional cerebral blood flow (rCBF), frequently employed in dementia assessment, reflects blood delivery to specific brain regions and serves as a sensitive functional biomarker of neurodegeneration (Tartaglia et al., 2011). Among functional modalities, SPECT is widely used to assess rCBF, providing an indirect measure of cerebral metabolic activity. Its advantages include lower cost, shorter acquisition times, and broader availability compared with PET, allowing detection of perfusion deficits even in structurally normal-appearing regions (Tartaglia et al., 2011; Swan et al., 2015; Ferrando and Damian, 2021). In SPECT, radiotracers such as technetium-99 m hexamethylpropylene amine oxime (^99m^Tc-HMPAO) cross the blood–brain barrier and are metabolized into a form that remains in neurons proportional to local rCBF at the time of injection, producing a static image of regional perfusion (Swan et al., 2015). SPECT images can then be analyzed to identify hypoperfusion, offering insights into functional integrity at both lobar and more localized levels.

In bvFTD, SPECT studies have consistently demonstrated bilateral yet asymmetric hypoperfusion, most pronounced in prefrontal and anterior temporal cortices, with a slight left-hemisphere predominance (Basely et al., 2013; Imokawa et al., 2024). Whole-brain analyses have further revealed extensive anterior hypoperfusion encompassing the frontal cortex, anterior cingulate, anterior temporal regions, and anterior parahippocampal/hippocampal structures, with relative sparing of occipital and parietal cortices (Basely et al., 2013). This anterior pattern has served as a key supportive feature for probable bvFTD diagnosis and helps differentiate bvFTD from Alzheimer’s disease (AD), in which posterior regions are predominantly affected (Valotassiou et al., 2012; Basely et al., 2013; Valotassiou et al., 2014; Imokawa et al., 2024). Nonetheless, hypoperfusion patterns have varied across individuals, occasionally extending to parietal regions, paralleling structural MRI findings and underlying neuropathological heterogeneity (Salmon et al., 2005; Cerami et al., 2016; Perry et al., 2017).

### Existing evidence on brain perfusion and clinical correlates in bvFTD

A growing body of literature has examined associations between rCBF abnormalities and clinical manifestations of bvFTD. From a cognitive perspective, previous SPECT studies have shown that cognitive deficits in bvFTD are closely associated with hypoperfusion in discrete brain regions. Specifically, executive function impairments - a hallmark of bvFTD neuropsychological profile - have been found to correlate with reduced perfusion in frontal, temporal, and parietal areas (Borroni et al., 2010). Memory impairments have been associated with hypoperfusion in medial temporal regions (Basely et al., 2013), language deficits with primarily left frontal and temporal areas, and visuospatial functions with parietal, precuneus, and cingulate regions (Borroni et al., 2010). Notably, global cognitive screening tools, such as the Mini-Mental State Examination (MMSE), have failed to demonstrate significant associations with brain perfusion in FTLD (Borroni et al., 2010).

Behavioral disturbances and neuropsychiatric symptoms have also been linked to specific regional perfusion abnormalities (Valotassiou et al., 2021a; Konstantinopoulou et al., 2024). Bilateral frontal hypoperfusion has been shown to correlate with the severity of behavioral disturbances, with greater reductions corresponding to more pronounced symptoms (Konstantinopoulou et al., 2024). Among these, apathy, one of the most frequent and disabling features of bvFTD, has been linked to reduced perfusion in bilateral frontal areas, bilateral anterior cingulate cortex, left posterior cingulate, and right temporal cortex (Valotassiou et al., 2020), with the anterior cingulate cortex consistently implicated in motivation and goal-directed behavior (Njomboro et al., 2012). Additionally, eating disorders, including hyperorality, have been linked to hypoperfusion in the right anterior and dorsolateral prefrontal cortices, left orbitofrontal cortex, orbital portion of the right inferior frontal gyrus, and left parahippocampal gyrus (Valotassiou et al., 2021b). Consistent with these findings, previous research has indicated that positive behavioral symptoms related to impulsivity and impaired inhibitory control, such as socially inappropriate behaviors, reflect orbitofrontal dysfunction, highlighting its critical role in emotion regulation and behavioral inhibition (Warriner and Velikonja, 2006; Torregrossa et al., 2008).

Disease severity has also been examined in relation to brain perfusion in bvFTD (Borroni et al., 2010; Konstantinopoulou et al., 2024; Chatzidimitriou et al., 2025a). Borroni et al. (2010) reported a significant association between greater disease severity and lower global rCBF in the frontal and temporal lobes bilaterally. Complementing these findings, Konstantinopoulou et al. (2024) observed correlations between disease severity and hypoperfusion in the bilateral frontal and temporal lobes, as well as in right limbic regions. More recently, Chatzidimitriou et al. (2025a) identified hypoperfusion in the right prefrontal and inferior parietal cortices as significant neural correlates of functional impairment in bvFTD. Interindividual differences in clinical severity have also been linked to the concept of brain reserve. Using SPECT imaging to assess rCBF, Borroni et al. (2009) demonstrated that bvFTD patients with higher educational or occupational attainment tolerate greater levels of brain dysfunction without manifesting more severe clinical symptoms, supporting the notion that cognitive reserve mitigates the functional consequences of underlying neuropathology.

Finally, despite the centrality of personality changes in bvFTD (Rascovsky et al., 2011), this domain remains largely unexplored in perfusion imaging research. Structural and metabolic studies have highlighted the involvement of medial prefrontal, orbitofrontal, and cingulate regions - areas critical for motivation, appropriate social behavior, and emotional regulation - in personality alterations (Warriner and Velikonja, 2006; Torregrossa et al., 2008; Njomboro et al., 2012). However, to our knowledge, no SPECT investigations to date have applied standardized personality inventories to directly examine the relationship between regional perfusion and personality changes in bvFTD, representing a significant gap, particularly as these alterations often constitute one of the earliest and most diagnostically distinctive features of the disease.

### Research gap and study goals

In summary, SPECT imaging has yielded valuable insights into the functional neuroanatomy of bvFTD; however, several critical gaps remain in understanding the precise relationship between rCBF abnormalities and the full spectrum of clinical manifestations in bvFTD. Most prior studies have examined brain dysfunction within large, functionally heterogeneous lobar regions, thereby limiting the ability to link specific regional abnormalities to distinct cognitive, behavioral, and socioemotional impairments. Moreover, the neural correlates of personality changes—a core and often early feature of bvFTD—remain largely unexplored in perfusion imaging research. Collectively, these gaps highlight the need for studies employing standardized, fine-grained anatomical approaches, such as Brodmann area (BA)-based analyses applied to SPECT data, to systematically investigate how localized perfusion deficits relate to the multifaceted clinical features of bvFTD.

This exploratory study aims to investigate the clinical correlates of brain hypoperfusion in bvFTD by employing ^99m^Tc-HMPAO SPECT imaging with automated BA analysis. Associations between regional cerebral perfusion abnormalities and cognitive deficits, behavioral disturbances, personality changes, and disease severity will be examined to provide a detailed functional neuroanatomical map of bvFTD symptomatology. By systematically linking BA-specific hypoperfusion to core clinical features, this study aims to elucidate the neurofunctional underpinnings of bvFTD’s heterogeneous phenotype, as well as inform the development of more targeted therapeutic interventions, ultimately improving personalized management and care for individuals affected by this complex and highly disabling clinical syndrome (Chatzidimitriou et al., 2023).

## Methods

### Participants

This prospective observational study enrolled 25 individuals diagnosed with at least possible bvFTD, according to the revised international behavioral variant FTDC criteria (Rascovsky et al., 2011). Patients and their caregivers were recruited from the 2nd Neurology Clinic of “AHEPA” University General Hospital of Thessaloniki, Greece, between January 2023 and November 2024. Diagnostic confirmation was established by a multidisciplinary team of neurologists and neuropsychologists, based on comprehensive clinical, neuropsychological, and neuroimaging evaluations, complemented by genetic and cerebrospinal fluid biomarker analyses to exclude alternative causes of dementia.

Eligible participants were required to have a close relative or friend familiar with their daily behavior and personality over time, capable of providing reliable information for caregiver-based assessments. To focus on individuals in the early stages of the disease, inclusion was limited to patients with a symptom duration of less than 3 years prior to enrollment, as determined through caregiver reports.

Patients were excluded if they presented with major vascular lesions on MRI or CT that could confound neuroimaging findings or if they had any other neurological disorders, such as stroke, Parkinson’s disease, multiple sclerosis, epilepsy, or a history of traumatic brain injury. Individuals with significant psychiatric conditions, including schizophrenia, bipolar disorder, or major depressive disorder, were also excluded. Finally, patients with severe sensory, motor, or physical impairments that could interfere with neuropsychological testing were considered ineligible to participate.

### Procedures

All participants were evaluated during routine outpatient visits at the Memory Clinic of “AHEPA” University Hospital. Assessments were carried out following standardized protocols to ensure procedural consistency and data reliability. Each patient completed the evaluation across two separate sessions (approximately 45 min each), scheduled in the morning hours to minimize fatigue and optimize performance. Testing took place in quiet, well-lit rooms, free from distractions, and included an extensive neuropsychological battery covering multiple cognitive domains.

Informant evaluations were conducted in a single structured session lasting approximately 2 hours, during which caregivers completed validated questionnaires assessing behavioral and personality changes, as well as disease severity.

All evaluations were administered by licensed clinical neuropsychologists with expertise in dementia assessment. The complete set of face-to-face and informant-based neuropsychological tools is described in detail in the sections below.

Brain perfusion was assessed using SPECT imaging, performed within a maximum of 2 weeks from the neuropsychological assessments of patients and their caregivers to ensure temporal consistency between measures.

### Ethical considerations

The study protocol was reviewed and approved by the Bioethics and Research Ethics Committee of the Aristotle University of Thessaloniki (AUTh), Greece (protocol code: 331191/2022, approval date: 20 December 2022). Written informed consent was obtained from all participants and their caregivers after a detailed explanation of the study’s objectives, procedures, and confidentiality safeguards. All personal data were anonymized and processed in accordance with the European Union General Data Protection Regulation (GDPR; Regulation (EU) 2016/679 of 27 April 2016). All research procedures adhered to the principles outlined in the Declaration of Helsinki (World Medical Association, 2024).

### Neuropsychological assessment

#### Global cognitive functioning

Overall cognitive status was evaluated using the Montreal Cognitive Assessment (MoCA) (Nasreddine et al., 2005), a brief screening tool designed to capture subtle cognitive changes. The MoCA was preferred over the MMSE because of its higher sensitivity to early deficits, particularly in executive domains that are often impaired in the initial stages of bvFTD. The validated Greek version was administered (Poptsi et al., 2019), and total scores (range: 0–30) were used in the analyses, with higher values indicating better cognitive performance.

#### Visuospatial abilities

Perceptual organization, visuospatial processing, and visuoconstructional skills were assessed using the Taylor Complex Figure Test (Taylor, 1959). Participants were instructed to reproduce a complex geometric figure as accurately as possible during the copy condition, within a 5-min time limit. Performance was scored based on the accuracy and spatial organization of 18 design elements, yielding a total score ranging from 0 to 36, which was included in the statistical analyses.

#### Memory

To assess long-term episodic verbal memory, the Story Memory Test from the “Neuropsychological Battery” (Kosmidis et al., 2011), developed by the Laboratory of Cognitive Neuroscience at the School of Psychology, AUTh, was administered. The Story Memory Test evaluates the capacity to encode and retrieve semantically related information. Participants heard a short story containing 16 informational units and were asked to recall it immediately and again after a 30-min delay filled with non-verbal tasks. For the purposes of this study, only the delayed recall score (range: 0–16) was analyzed, providing an index of verbal episodic long-term memory.

#### Executive functioning

Executive abilities were assessed using both traditional and computerized measures. The Greek version of the Trail Making Test - Part B (TMT-B) (Reitan, 1958; Zalonis et al., 2008) was administered to evaluate cognitive flexibility and set-shifting abilities. Participants were instructed to connect numbers and letters in alternating ascending order (1-A-2-B, etc.) as quickly and accurately as possible. The completion time in seconds served as the performance score, with shorter times reflecting better executive control.

Additionally, a computerized assessment of executive functioning was conducted using the REMEDES for Alzheimer-Revised (R4Alz-R) battery (Poptsi et al., 2024a; Poptsi et al., 2024b), a digital tool designed to evaluate core components of cognitive control. The R4Alz-R was selected for its ecological validity, sensitivity to executive dysfunction, and minimal cultural or educational bias. Three modules were administered: (i) Working Memory Capacity, including short-term storage, processing, and updating subtests; (ii) Attentional Control, assessing sustained, selective, and divided attention; and (iii) Executive Functioning, including inhibition and task/rule switching subtests. Performance scores from each module were standardized (z-scores) and combined to yield a total composite score, with higher values indicating poorer performance.

#### Social cognition

Theory of mind and social inferencing abilities were measured using the Greek adaptation of the Awareness of Social Inference Test - Short (TASIT-S) (McDonald et al., 2017; Tsentidou et al., 2021). Specifically, Part 2 of the test, the Test of Social Inference—Minimal (SI-M), was administered to evaluate participants’ capacity to infer others’ mental states and communicative intentions. The SI-M consists of brief video vignettes depicting both sincere and sarcastic social exchanges, requiring participants to interpret subtle verbal and nonverbal cues such as facial expressions, gestures, and tone of voice. After viewing each of the nine scenarios, participants answered “yes/no” questions regarding the characters’ actions, statements, thoughts, and feelings. Total scores ranged from 0 to 36, reflecting the number of correct responses, with higher values indicating better social inferencing abilities.

#### Personality assessment

Personality characteristics were evaluated using the Greek adaptation of Goldberg’s International Personality Item Pool (IPIP) Big-Five Questionnaire (Goldberg et al., 2005; Ypofanti et al., 2015). This 50-item measure assesses five major personality dimensions in accordance with the Big Five theory of personality (Costa and McCrae, 1992): Extraversion, Agreeableness, Conscientiousness, Emotional Stability-Neuroticism, and Intellect/Openness to Experience, rated on a 5-point Likert scale (1 = very inaccurate to 5 = very accurate). Given the reduced insight typical of bvFTD (Bracca et al., 2024), all questionnaire items were rephrased in third-person form (“he/she”) and completed by knowledgeable informants familiar with the patient’s long-term behavior and personality. Caregivers completed the questionnaire twice: first by retrospectively rating the patient’s premorbid personality (approximately 20 years prior to symptom onset), and second by rating the patient’s current personality traits following disease onset. Composite scores were calculated for each personality dimension (range: 10–50), and difference scores between premorbid and current ratings were computed for all five traits. These difference scores, reflecting the magnitude of disease-related longitudinal personality change, were included in the analyses of the present study.

#### Behavioral assessment

Behavioral symptoms characteristic of bvFTD were quantified using the Greek version of the Frontal Behavioral Inventory (FBI) (Kertesz et al., 1997; Konstantinopoulou et al., 2012), a 24-item informant-based questionnaire specifically designed to capture behavioral changes associated with FTD. The FBI includes two subscales assessing (i) negative symptoms associated with executive and motivational deficits (e.g., apathy, inflexibility, loss of insight), and (ii) positive symptoms reflecting behavioral disinhibition (e.g., impulsivity, social inappropriateness, hyperorality). Each item is rated on a 4-point scale (0 = none to 3 = severe), resulting in two subscale scores ranging from 0 to 36. Higher scores indicate more pronounced behavioral disturbances.

#### Disease severity

Disease stage and overall functional impairment were assessed with the Greek version of the Frontotemporal Dementia Rating Scale (FRS) (Mioshi et al., 2010; Maiovis et al., 2016) a staging instrument specifically developed for FTD. The FRS includes 30 items evaluating both behavioral and functional domains. After scoring, the raw total is divided by the number of applicable items and multiplied by 100 to derive a percentage score. Higher FRS percentages reflect milder disease severity and better overall functioning.

Disease severity was also assessed using the Clinical Dementia Rating (CDR) scale (Morris, 1993), which evaluates cognitive and functional performance across six domains: memory, orientation, judgment and problem solving, community affairs, home and hobbies, and personal care. The CDR yields both a Global Score (CDR-GS; 0–3) and a Sum of Boxes score (CDR-SB; 0–18). To enhance sensitivity to FTLD-specific symptomatology, the FTLD-Modified CDR (FTLD-CDR) (Knopman et al., 2008) was also employed, adding two additional domains - language and behavior - and providing a composite Sum of Boxes score (0–24). Higher scores indicate greater overall impairment.

### Neuroimaging assessment: brain perfusion SPECT scans

Brain perfusion imaging data were acquired at the Second Academic Nuclear Medicine Department of “AHEPA” University General Hospital of Thessaloniki, Greece. rCBF was measured using SPECT imaging to evaluate perfusion patterns across cerebral lobes and BAs. All imaging procedures including patient preparation, data acquisition, and processing followed the European Association of Nuclear Medicine (EANM) guidelines for brain perfusion SPECT studies (Kapucu et al., 2009), ensuring methodological consistency and optimal data quality. Prior to scanning, participants were instructed to avoid alcohol, caffeine, and other substances that could affect rCBF. SPECT scans were performed 30 min after intravenous administration of 740 MBq of 99mTc-HMPAO, with participants at rest, eyes open, and ears unplugged, in a quiet room to minimize external stimuli. A single-headed gamma camera (Philips, with Pegasys processor) was used to acquire 120 projections per participant over a total scan duration of 40 min (20 s per projection). Image reconstruction was performed on the XELERIS Workstation (Version 3.1) using filtered back projection with a Butterworth filter (order 10, cutoff 0.5), and images were reoriented in coronal, sagittal, and transverse planes. Automated analysis of SPECT data was conducted using NeuroGam™ software (GE Medical Systems, Segami Corporation, Columbia, South Carolina, USA), allowing quantification of rCBF in lobes and BAs across both hemispheres. The software aligns images within a standardized reference space based on Talairach’s atlas, allowing comparisons between individuals irrespective of variations in brain size, shape, or positioning. NeuroGam™ further enables comparison with an age-adjusted normative database, facilitating the detection of perfusion abnormalities. In this study, rCBF values were converted to z-scores relative to an age-matched control group, accounting for age-related effects and ensuring comparability across participants.

### Data analysis

Standardized rCBF values, expressed as z-scores, were derived from the NeuroGam™ software (GE Medical Systems, Segami Corporation, Columbia, SC, USA) for each participant. Perfusion values were analyzed at both the lobar (frontal, temporal, limbic, parietal, and occipital) and BA levels. To focus on the most clinically and functionally relevant brain regions, only BAs exhibiting hypoperfusion greater than 3 standard deviations below the normative mean in more than 50% of the sample were retained for further analyses. This approach also served as a dimensionality reduction strategy, limiting the number of variables included in the analyses and ensuring sufficient statistical power by focusing on the most severely affected areas.

Associations between rCBF in lobes or selected BAs and clinical variables were examined using non-parametric Spearman correlations conducted in IBM SPSS Statistics, Version 27 (IBM Corp., Armonk, NY, USA). To control the family-wise error rate (FWER) and reduce the risk of Type I errors arising from multiple comparisons, an appropriate multiple testing correction method was applied.

Specifically, the analytical design of this study included 14 clinical variables and 10 BAs, resulting in a total of 140 correlation tests (14 clinical variables x 10 brain regions). While a conventional Bonferroni correction across all 140 tests would formally control the FWER, such an approach would be overly conservative, yielding a very stringent significance threshold and substantially reducing sensitivity for detecting statistically significant associations. Given the expected non-independence among both the clinical variables and the BA-level rCBF measures, we therefore employed a correction approach based on the effective number of independent tests (Meff) to adjust the Bonferroni correction factor accordingly.

The estimation of Meff was performed in the R statistical programming and computing environment (version 4.5.2; R Foundation for Statistical Computing, Vienna, Austria; www.r-project.org; R Core Team, 2025) using the “meff()” function from the “poolr” package (Cinar and Viechtbauer, 2022). This approach estimates the number of statistically independent tests by leveraging the eigenvalue structure of the correlation matrix, thereby explicitly accounting for the underlying correlation structure among variables. Four established methods implemented in “poolr” were applied (Nyholt, 2004; Li and Ji, 2005; Gao et al., 2008; Galwey, 2009). Across these approaches, the estimated effective number of independent tests ranged from 21 to 24, reflecting the considerable correlation among both clinical and brain measures. We selected the Li and Ji (2005) method, which is widely used and regarded as a balanced and conservative estimator in the presence of correlated tests, yielding an effective number of independent tests of 22.

Using this value, a Bonferroni-style correction was applied, resulting in an adjusted significance threshold of: α_corrected_ = 0.05/22 ≈ 0.0023. All correlation analyses were subsequently evaluated for statistical significance using this adjusted threshold.

## Results

### Demographic and clinical characteristics of participants

The study included 25 individuals meeting diagnostic criteria for bvFTD (Rascovsky et al., 2011), comprising 12 men and 13 women. Participants had a mean age of 70.20 years (SD = 7.72), with an average age at symptom onset of 68.10 years (SD = 7.85). The cohort demonstrated a mean educational attainment of 10.65 years (SD = 3.84). Among the sample, two female patients carried a genetically confirmed C9orf72 expansion.

Clinical assessments indicated a mean Clinical Dementia Rating Scale (CDR) Global Score of 1.17 (SD = 0.68) and a mean CDR Sum of Boxes of 6.60 (SD = 3.88), consistent with mild dementia severity. As expected, the FTLD-modified CDR Sum of Boxes yielded higher values (M = 8.60, SD = 4.41), capturing deficits characteristic of FTD. The mean performance on the MoCA test was 15.31 (SD = 4.98), reflecting substantial decline in global cognition. An overview of participants’ demographic and clinical features is provided in Table 1.

**Table 1:** Demographic and clinical characteristics of participants.

bvFTD patients	Mean (SD)
n	25
Age, years	70.20 (7.72)
Age at onset, years	68.10 (7.85)
Sex, M/F, n	12/13
Education, years	10.65 (3.84)
CDR global score	1.17 (0.68)
CDR sum of boxes	6.60 (3.88)
FTLD-CDR sum of boxes	8.60 (4.41)
MoCA	15.31 (4.98)

#### Selection of brain areas based on Hypoperfusion

Of all measured lobes and BAs, only those demonstrating hypoperfusion greater than 3 standard deviations below the mean, compared to normative data, were retained for further analysis. These areas included: BA 5 R, BA 6 L, BA 8 R, BA 11 R, BA 28 L, BA 31 L, BA 32 R, BA 37 L, BA 40 R, and BA 46 R.

#### Associations between rCBF and behavioral disturbances

Statistically significant negative correlations were identified between FBI negative symptoms and rCBF in the right BA 8 (*r* = −0.469, *p* = 0.002). Additionally, FBI positive symptoms showed statistically significant negative correlations with rCBF in the right BA 46 (*r* = −0.506, *p* = 0.002).

#### Associations between rCBF and personality traits

Correlational analyses were performed to examine the relationship between brain perfusion and personality changes in individuals with bvFTD. Statistically significant negative correlations were identified between the magnitude of decline in conscientiousness and rCBF in the left BA 6 (*r* = −0.541, *p* = 0.001) and in the left BA 31 (*r* = −0.571, *p* = 0.001). Additionally, statistically significant negative correlations were identified between the magnitude of decline in extraversion and rCBF in the right BA 8 (*r* = −0.532, *p* = 0.002), in the right BA 32 (*r* = −0.643, *p* = 0.001), and in the left BA 37 (*r* = −0.443, *p* = 0.002). No other relationships between personality traits and rCBF survived Bonferroni correction for multiple comparisons.

#### Associations between rCBF and cognitive deficits

The MoCA test did not demonstrate any statistically significant correlations with rCBF in any of the brain lobes or specific BAs.

The copy condition of the Taylor Complex Figure Test demonstrated a significant positive correlation with rCBF in the right BA 5 (*r* = 0.408, *p* = 0.002).

The delayed recall of the Story Memory test showed significant positive correlations with rCBF in the left BA 28 (*r* = 0.521, *p* = 0.001) and left BA 31 (*r* = 0.475, *p* = 0.002).

Performance on the TMT-Part B showed significant negative correlations with rCBF in the left BA 6 (*r* = −0.527, *p* = 0.001) and left BA 31 (*r* = −0.531, *p* = 0.001).

Additionally, the R4Alz-R Battery total score showed significant negative correlations with rCBF in the right BA 5 (*r* = −0.472, *p* = 0.002) and the right BA 8 (*r* = −0.565, *p* = 0.001).

Finally, the TASIT-S Part 2 demonstrated significant positive correlations with rCBF in the right BA 11 (*r* = 0.461, *p* = 0.002), right BA 32 (*r* = 0.431, *p* = 0.002), left BA 37 (*r* = 0.561, *p* = 0.001), right BA 40 (*r* = 0.471, *p* = 0.002), and right BA 46 (*r* = 0.442, *p* = 0.002).

#### Associations between rCBF and disease severity

Correlational analyses were performed to examine the relationship between rCBF and the FRS scale. Significant positive associations were identified between the FRS percentage and rCBF in the right BA 8 (*r* = 0.490, *p* = 0.002).

An overview of the statistically significant associations between rCBF and clinical variables is presented in Table 2. Results for non-significant relationships between lobar rCBF and clinical variables are reported in Supplementary material.

**Table 2:** Statistically significant correlations between rCBF and various clinical variables in early-stage bvFTD (Bonferroni corrected).

Variable	BA	*r*	*p*
Disease severity			
FRS percentage	BA 8 R	0.490	0.002
Behavioral disturbances			
FBI negative symptoms	BA 8 R	−0.469	0.002
FBI positive symptoms	BA 46 R	−0.506	0.002
Personality changes			
Decline in conscientiousness	BA 6 L / BA 31 L	−0.541 / −0.571	0.001/0.001
Decline in extraversion	BA 8 R / BA 32 R / BA 37 L	−0.532 / −0.643 / −0.443	0.002/0.001/0.002
Decline in agreeableness	-		
Decline in emotional stability	-		
Decline in intellect	-		
Cognitive measures			
MoCA	-		
Taylor—copy condition	BA 5 R	0.408	0.002
Story memory	BA 28 L / BA 31 L	0.521/0.475	0.001/0.002
TMT-Part B	BA 6 L / BA 31 L	−0.527 / −0.531	0.001/0.001
R4Alz-R battery	BA 5 R / BA 8 R	−0.472 / −0.565	0.002/0.001
TASIT-S Part 2	BA 11 R / BA 32 R / BA 37 L / BA 40 R / BA 46 R	0.461/0.431/0.561/0.471/0.442	0.002/0.002/0.001/0.002/0.002

**Figure 1:**
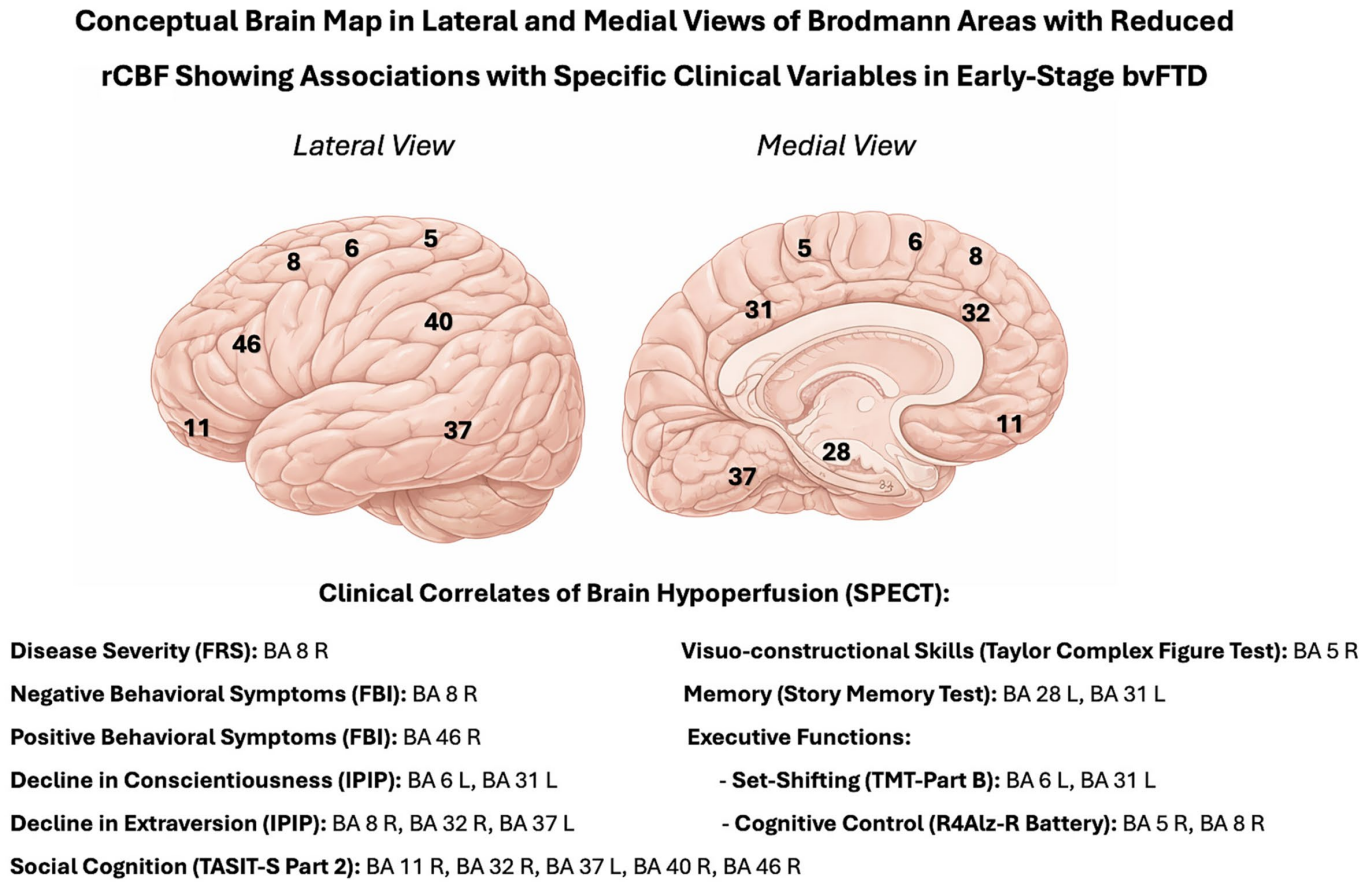
Conceptual brain map illustrating BAs with reduced rCBF and their associations with clinical variables in early-stage bvFTD. BA, Brodmann area; FBI, Frontal Behavioral Inventory; FRS, Frontotemporal Dementia Rating Scale; IPIP, Goldberg’s International Personality Item Pool Big-Five Questionnaire; L, left hemisphere; R, right hemisphere; rCBF, regional cerebral blood flow; TASIT-S, The Awareness of Social Inference Test - Short Version; TMT, Trail Making Test.

In addition, a conceptual schematic brain map illustrating the BAs showing statistically significant correlations with specific clinical variables is presented in Figure 1, offering a visual overview of the key brain-behavior associations identified in the study.

## Discussion

The present study systematically examined the rCBF correlates of cognitive, behavioral, personality, and functional features in individuals with bvFTD using 99mTc-HMPAO SPECT imaging analyzed at the BA level. The main aim was to clarify how regional perfusion patterns relate to the diverse clinical manifestations of bvFTD, thereby providing a more anatomically specific understanding of its core clinical features. The results revealed several robust correlations between rCBF in discrete cortical regions and distinct clinical parameters, indicating that variations in localized perfusion may reflect the regional neurobiological substrates of bvFTD symptomatology. Overall, these findings indicate that interindividual differences in the clinical presentation of bvFTD may correspond to distinct perfusion profiles across key brain regions.

### Clinical correlates of regional brain perfusion in bvFTD

#### Global cognition

The absence of significant correlations between global cognitive performance and rCBF is consistent with previous SPECT and PET studies indicating that global indices such as the MMSE or MoCA are relatively insensitive to the anteriorly distributed dysfunction typical of bvFTD (Borroni et al., 2010; Deutsch et al., 2016). These screening measures predominantly assess posterior cortical functions - orientation, visuospatial ability, and language - that tend to be relatively preserved in the early stages of bvFTD. The lack of association in the current results therefore reflects both the selectivity of bvFTD-related frontal dysfunction and the limitations of global tests for capturing the subtle yet clinically significant impairments in executive and socioemotional domains.

#### Visuospatial and Visuoconstructional functioning

A positive correlation between visuoconstructional abilities and rCBF in a right parietal region (BA 5) highlights the essential contribution of this area to spatial integration and visuomotor organization in bvFTD. BA 5, located in the superior parietal lobule, is crucial for coordinating spatial and motor representations required for complex figure construction (Vandenberghe and Gillebert, 2009). This region also maintains functional connections with frontal executive areas within the frontoparietal control network, which supports higher-order planning, organization, and strategy use (Vandenberghe and Gillebert, 2009). The observed hypoperfusion in BA 5 may therefore reflect both posterior cortical involvement and disrupted frontoparietal interactions, leading to impaired integration of spatial and executive components necessary for effective visuoconstructional processing.

#### Memory functioning

Memory performance, as assessed by delayed recall of a Story Memory test, was positively associated with rCBF in left BAs 28 and 31, corresponding, respectively, to the parahippocampal region and posterior cingulate cortex. These structures are integral to the retrieval and contextualization of episodic memories (Aminoff et al., 2013; Leech and Sharp, 2013). While bvFTD is not classically characterized by primary memory deficits, dysfunction in medial temporal and limbic regions has been reported to contribute to episodic memory impairments (Perry et al., 2017). The parahippocampal cortex is critically involved in encoding and retrieval of contextual and spatial aspects of episodic memory, supporting the integration of information into coherent memory traces (Aminoff et al., 2013). Additionally, the posterior cingulate cortex has been linked to self-referential processing and autobiographical recall (Leech and Sharp, 2013), and its involvement here may partly reflect the nature of the memory assessment used in this study - a story-based test that might have unconsciously led participants to relate the material to their personal experiences.

#### Executive functioning

Executive performance correlated with perfusion in both left and right frontal and parietal regions, including the bilateral frontal cortex (BAs 6 and 8), left posterior cingulate (BA 31), and right parietal (BA 5). These areas are critically implicated in planning, task switching, and response monitoring (Stuss and Alexander, 2007; Dadario et al., 2023). Notably, hypoperfusion in the right BA 8, located in the superior prefrontal gyrus just anterior to the premotor cortices, has been associated with decision-making under uncertainty, oculomotor control, motor planning, selection among competing stimuli, and adaptive cognitive control (Dadario et al., 2023). Additionally, reduced perfusion in the left premotor region (BA 6), situated anterior to the primary motor cortex, may reflect impairments in the generation, sequencing, and execution of goal-directed actions, a deficit frequently observed in bvFTD. The associations involving BA 31 further underscore the role of cingulate regions in sustaining attentional control and performance monitoring during complex cognitive tasks (Leech and Sharp, 2013). Interestingly, although involvement of BA 32 - a key node of the cingulo-opercular action-mode network implicated in executive control (Dosenbach et al., 2025)—swas anticipated, we did not observe statistically significant associations in our cohort. This absence may reflect the modest sample size, heterogeneity in perfusion patterns, or task-specific functional engagement, and does not preclude a role for BA 32 in supporting sustained goal-directed attention and executive control. Taken together, these results indicate that hypoperfusion in distributed but regionally specific frontal and parietal cortices is linked to poorer executive performance in bvFTD. These findings are also in line with prior SPECT studies reporting correlations between executive measures and rCBF in frontal and parietal regions (Borroni et al., 2010), reinforcing the view that executive dysfunction in bvFTD reflects regionally localized frontal and parietal hypoperfusion, but also potential disruption of frontoparietal functional systems supporting higher-order cognitive control.

#### Socioemotional cognition

The most extensive brain-behavior associations were observed for social cognition, which correlated with rCBF in several regions including the right orbitofrontal cortex (BA 11), right anterior cingulate (BA 32), left inferior temporal area (BA 37), right inferior parietal cortex (BA 40), and right dorsolateral prefrontal cortex (BA 46). Each of these regions has been independently implicated in core social cognitive processes such as theory of mind, perspective taking, sarcasm perception, emotional evaluation, and empathic processing (Frith and Frith, 2006; Rankin et al., 2009; Ibañez and Manes, 2012). The orbitofrontal cortex (BA 11) plays a central role in evaluating the emotional and reward-related value of social stimuli and in guiding socially appropriate decision-making (Rolls, 2004). Hypoperfusion in this region has been consistently associated with impaired emotional regulation, altered social judgment, and disinhibited behavior in bvFTD (Rolls, 2004). Moreover, the involvement of the right dorsolateral prefrontal cortex (BA 46) highlights the contribution of executive control mechanisms to social cognition, including the regulation of social responses, maintenance of social rules, and flexible adjustment of behavior in dynamic interpersonal contexts (Miller and Cohen, 2001; Rankin et al., 2009). Similarly, the anterior cingulate (BA 32) is critically involved in affective monitoring and modulation, motivational drive, and the integration of emotional signals with goal-directed behavior (Seeley et al., 2007; Njomboro et al., 2012; Leech and Sharp, 2013). Dysfunction within this region has been linked to apathy, reduced empathy, and diminished social engagement in FTLD syndromes (Seeley et al., 2007; Njomboro et al., 2012; Leech and Sharp, 2013). Beyond frontal and cingulate regions, significant associations were also identified in temporal and parietal cortices. The inferior temporal cortex (BA 37) contributes to high-level visual and semantic processing of socially relevant stimuli, including facial identity and emotional expressions, while the inferior parietal cortex (BA 40) supports the integration of multimodal contextual and somatosensory information necessary for inferring others’ mental states (Saxe and Kanwisher, 2003; Olson et al., 2007). The observed correlations between TASIT-S performance and rCBF in these regions suggests that effective social inference relies on intact perceptual, semantic, executive, and contextual processing, which may become compromised as perfusion declines. Although causal interpretations cannot be drawn from the present correlational design, the observed pattern indicates that reduced perfusion across discrete but interconnected frontal, temporal, limbic, and parietal regions may underlie the complex social cognitive deficits that represent a core and predictive clinical hallmark of bvFTD (Chatzidimitriou et al., 2025b).

#### Behavioral disturbances

Behavioral disturbances in bvFTD were significantly associated with lower perfusion in right prefrontal areas, specifically BAs 8 and 46. This pattern is consistent with prior findings linking right-sided frontal hypoperfusion to behavioral disinhibition, apathy, and loss of social insight (Warriner and Velikonja, 2006; Konstantinopoulou et al., 2024). Negative behavioral symptoms, such as apathy and emotional flatness, correlated with perfusion in BA 8, a region implicated in sustained attention, initiation of goal-directed behavior, and adaptive cognitive control (Dadario et al., 2023). Hypoperfusion in this area may reflect a diminished capacity to initiate and maintain goal-directed activity, contributing to the passivity and motivational deficits frequently observed in bvFTD (Chatzidimitriou et al., 2025a). In contrast, positive behavioral symptoms, including impulsivity and disinhibition, correlated with perfusion in BA 46, a region encompassing the dorsolateral prefrontal cortex, which supports inhibitory control and behavioral modulation. These findings suggest that different aspects of behavioral dysregulation in bvFTD may be associated with dysfunction in distinct prefrontal regions, with right BA 8 contributing primarily to motivational drive and initiation of goal-directed actions, and right BA 46 supporting behavioral control. Overall, the current results align with previous SPECT and PET studies identifying right frontal dysfunction as a prominent neural correlate of both apathic and disinhibited behavioral phenotypes in bvFTD (Torregrossa et al., 2008; Valotassiou et al., 2020; Konstantinopoulou et al., 2024).

#### Personality correlates

A particularly novel contribution of this study is the identification of specific rCBF correlates of disease-related personality changes in bvFTD, a domain rarely examined in brain perfusion imaging studies. In the present cohort, changes in conscientiousness showed positive associations with perfusion in the left premotor cortex (BA 6) and left dorsal posterior cingulate cortex (BA 31), whereas changes in extraversion correlated with perfusion in a right prefrontal region (BA 8), right dorsal anterior cingulate (BA 32), and left temporal cortex (BA 37).

The link between conscientiousness and perfusion in premotor and cingulate regions is consistent with the role of these areas in the planning and regulation of purposeful behavior (Tanaka et al., 2005; Leech and Sharp, 2013). Conscientiousness involves self-discipline, orderliness, dutifulness and goal maintenance, characteristics that depend on intact premotor and cingulate function (Costa and McCrae, 1992; Wilson et al., 2015). Specifically, the premotor cortex (BA 6) is critical for planning, initiating, and executing purposeful actions, supporting structured, goal-directed behavior. In addition, BA 6 contributes to attentional control and the temporal maintenance of task-relevant information in working memory, integrating spatial and verbal representations necessary for achieving complex goals and guiding adaptive behavior (Tanaka et al., 2005). On the other hand, the dorsal posterior cingulate cortex (BA 31) is traditionally implicated in self-referential processing and monitoring ongoing actions, integrating internal goals with external demands, and aligning behavior with current tasks and long-term objectives (Leech and Sharp, 2013). Importantly, this region is a key node of the default mode network, showing increased activity during autobiographical memory retrieval, future planning, and internally-directed cognition, suggesting a central role in regulating attention toward internally relevant goals. Together, these regions facilitate goal-directed, adaptive self-regulation (Leech and Sharp, 2013). Prior studies have suggested that higher levels of conscientiousness may act as a protective factor associated with better cognitive and functional trajectories in later life (Wilson et al., 2015); the current findings provide preliminary evidence of possible regional substrates underlying this effect.

Extraversion change correlated positively with perfusion in right prefrontal cortex (BA 8), right dorsal anterior cingulate (BA 32), and left temporal cortex (BA 37). The right prefrontal region (BA 8) plays a critical role in executive control, attentional shifting, and the organization of goal-directed behavior (Dadario et al., 2023). Activity within this region supports the initiation and maintenance of actions in response to motivationally relevant environmental cues and facilitates adaptive decision-making in uncertain situations, such as socially complex contexts. The dorsal anterior cingulate cortex is involved in emotional engagement, reward processing, and motivational drive (Bush et al., 2002), processes that underlie sociability and approach behavior. The left temporal cortex, encompassing BA 37, includes the fusiform and inferior temporal gyri, which are critical for high-level visual processing (Ardila et al., 2015), including facial recognition and the interpretation of socially relevant visual cues. Adjacent parahippocampal regions also contribute to contextual and mnemonic aspects of social interactions (Aminoff et al., 2013), by linking social experiences to memory and emotional context. Hypoperfusion in these areas may therefore contribute to the blunted affect, social withdrawal, reduced motivation, and impaired interpersonal processing frequently observed in individuals with bvFTD.

Overall, these findings provide novel SPECT-based evidence linking regional brain perfusion to the magnitude of personality change in bvFTD, highlighting the functional neuroanatomical substrates underlying disease-related alterations in conscientiousness and extraversion. The integration of frontal executive, cingulate evaluative, and temporal mnemonic processes appears essential for maintaining adaptive personality features, and disruption of these regions may help explain the pronounced personality changes characteristic of bvFTD. It is recommended that future research examine personality-related perfusion correlates in other clinical populations as well as in healthy adults, to more clearly delineate the neural underpinnings of personality traits and clarify whether these associations are disease-specific or reflect broader brain-personality relationships.

#### Disease severity

Disease severity, as assessed by the FRS, was positively associated with perfusion in the right superior prefrontal cortex (BA 8). This result indicates that higher perfusion in this region corresponds to less functional impairment. The right superior frontal cortex has been implicated in strategic planning and sustained attention (Dadario et al., 2023), abilities essential for maintaining independence in daily life. Prior SPECT studies have similarly linked disease severity and functional decline in bvFTD to reduced perfusion in frontal areas (Borroni et al., 2009; Konstantinopoulou et al., 2024; Chatzidimitriou et al., 2025a). The current finding reinforces the importance of prefrontal integrity for global clinical functioning and suggests that perfusion in this region could serve as a potential biomarker of disease progression.

#### Strengths and limitations

The present study employed a fine-grained, BA-based analysis of SPECT data in combination with a comprehensive clinical characterization, encompassing cognitive, behavioral, personality, and disease-severity measures. This integrative approach enabled a multidimensional examination of brain-behavior relationships in bvFTD and allowed for anatomically specific associations between rCBF and distinct clinical features. By moving beyond global cognitive indices and focusing on domain-specific clinical correlates, this study provides a more nuanced understanding of the functional neuroanatomy underlying the heterogeneous clinical presentation of bvFTD.

However, several limitations should be acknowledged. First, the relatively small sample size, although comparable to that of other neuroimaging studies in bvFTD, significantly limits the generalizability of the findings and may reduce statistical power. Additionally, the cross-sectional and correlational nature of the design precludes causal inferences regarding the directionality of the observed associations; consequently, the reported relationships should be interpreted as indicative rather than explanatory. Longitudinal studies are required to determine whether regional perfusion alterations precede, accompany, or follow clinical deterioration and to clarify their potential role as biomarkers of disease progression.

Moreover, in this study, disease severity and early-stage characterization were determined exclusively based on caregiver-reported estimates of symptom duration, which are inherently prone to bias. Standardized clinical staging criteria, such as the CDR score, were not employed for eligibility, which limits the precision of classifying disease stage. Consequently, statements referring to “early-stage” bvFTD should be interpreted with caution, and future studies would benefit from incorporating formalized staging criteria to reduce subjectivity and improve reproducibility.

Additionally, although this study provides new insights into the neural correlates of personality changes in bvFTD, it is important to note that these changes were assessed retrospectively using caregiver reports referencing a 20-year premorbid interval, which may be subject to recall bias. There are currently no established paradigms for such assessments in bvFTD research, making this approach novel. The extended premorbid timeframe was chosen to minimize the likelihood that subtle, prodromal behavioral changes influenced caregiver ratings. Nevertheless, this retrospective method is inherently prone to memory inaccuracies (including potential halo-horn effects), which may artificially inflate observed correlations. Future studies would benefit from exploring different premorbid reference windows, implementing improved anchoring strategies, or incorporating complementary objective measures to enhance the reliability, validity, and objectivity of premorbid personality assessments in bvFTD.

Another considerable methodological limitation of this study is the selection of BAs based on hypoperfusion greater than 3 standard deviations in at least 50% of the sample. While this approach allowed us to focus on the most severely affected and clinically relevant regions and to ensure adequate statistical power, it may have excluded areas with subtler perfusion changes that could also contribute to cognitive, behavioral, or personality alterations. Future studies with larger samples could explore more liberal thresholds or alternative dimensionality reduction strategies.

Furthermore, an additional methodological limitation of the present study relates to the exclusive use of functional imaging data. While brain perfusion SPECT is sensitive to early functional changes and remains a widely accessible tool in clinical settings, it provides an indirect measure of neuronal activity that offers lower spatial resolution than other neuroimaging modalities, such as PET, which may constrain precise anatomical localization. In addition, partial volume correction was not applied in the imaging pipeline, and regional differences in cortical atrophy were not explicitly accounted for, which may complicate the interpretation of perfusion measures and potentially confound brain-behavior associations in a neurodegenerative population.

Moreover, the absence of complementary structural neuroimaging data, such as MRI-derived volumetric measures, has limited the ability to directly relate functional perfusion abnormalities to underlying patterns of cortical and subcortical atrophy. The integration of multimodal neuroimaging approaches - combining functional techniques such as SPECT with structural modalities like MRI - could have offered a more comprehensive characterization of the neurobiological substrates of bvFTD. Such approaches are particularly valuable for disentangling the relative contributions of structural degeneration and functional abnormalities to clinical symptomatology and for capturing complementary aspects of brain organization and pathology. Future studies would therefore benefit from leveraging multimodal imaging data to achieve a deeper and more integrative understanding of brain-behavior relationships in bvFTD.

Finally, the present study focused only on region-specific associations between rCBF and clinical measures. Beyond region-specific analyses, future research using brain SPECT imaging data may benefit from adopting network-level analytical approaches to better capture large-scale patterns of functional organization in bvFTD. Multivariate, data-driven techniques applied to rCBF measures - such as dimensionality reduction analyses - could help identify coordinated perfusion patterns across distributed brain regions, thereby providing valuable insights into the functional networks underlying cognitive, behavioral, and personality changes in bvFTD. Such approaches may be particularly informative in larger samples, where greater statistical power would facilitate the identification of stable network-level signatures. Leveraging SPECT imaging in this way could extend its utility beyond regional characterization, potentially offering a complementary perspective on the network-level functional alterations that characterize bvFTD.

### Clinical implications

By identifying region-specific associations between rCBF and key clinical domains, this study advances the functional characterization of bvFTD. The findings suggest that regional perfusion mapping can yield clinically meaningful information, particularly in early-stage or atypical presentations that may appear structurally normal. From a clinical perspective, understanding which cortical regions are associated with specific symptoms may assist in the development of targeted interventions. Importantly, the identification of regionally specific perfusion correlates also opens avenues for neuromodulatory and pharmacological strategies aimed at restoring or compensating for regional dysfunction. Emerging interventions such as transcranial magnetic stimulation (TMS), transcranial direct current stimulation (tDCS), or focused drug delivery could be directed toward the cortical regions implicated in clinical symptoms (Masson-Trottier et al., 2025). Such approaches might modulate neuronal excitability or neurotransmitter release within hypoperfused areas, thereby potentially mitigating core clinical features of bvFTD. While preliminary, these findings can provide a functional map that could serve as a basis for individualized, region-targeted treatment planning in future clinical trials. These directions highlight the translational potential of regional perfusion imaging not only as a diagnostic and prognostic biomarker but also as a guide for developing precision-based neuromodulatory and rehabilitative interventions in bvFTD. Furthermore, the demonstration of personality-related perfusion correlates offers new insights into the biological basis of personality changes in bvFTD, potentially informing early diagnostic markers and caregiver counseling.

Future studies are encouraged to adopt a comprehensive, multi-modal approach by integrating SPECT with other structural (e.g., MRI) or metabolic (e.g., FDG-PET) imaging techniques. Such designs can provide a deeper understanding of how brain dysfunction relates to specific cognitive, behavioral, and personality changes, thereby elucidating the complex neurobiological mechanisms underlying bvFTD. In addition, longitudinal follow-up will be crucial to determine whether baseline perfusion in specific brain regions predicts subsequent symptom progression, informing both prognosis and care planning. Finally, research in larger, longitudinal cohorts is warranted to validate the observed brain-behavior associations and to evaluate the potential utility of the identified BAs as biomarkers for monitoring disease progression and assessing the efficacy of implemented therapeutic interventions.

### Conclusion

In conclusion, this study demonstrates that regional alterations in cerebral perfusion are associated with the diverse clinical features of bvFTD. Distinct brain regions appear to be linked to specific cognitive, behavioral, personality, and functional parameters, reflecting the multifaceted nature of the syndrome. Although cross-sectional and correlational in design, these findings provide an anatomically specific overview of the clinical-perfusion relationships observable with SPECT imaging. The inclusion of personality correlates also represents a particularly novel aspect of this study, emphasizing that personality changes in bvFTD are associated with identifiable regional perfusion deficits. Further research in larger and longitudinal cohorts is needed to validate these associations and to explore the potential of the identified BAs as biomarkers for symptom monitoring and personalized clinical management.

## Data Availability

The raw data supporting the conclusions of this article will be made available by the authors, without undue reservation.
