# Supplementary material for: Mapping the clinical correlates of brain hypoperfusion in behavioral variant frontotemporal dementia: insights from a SPECT imaging study
Source: Front Neurosci. 2026 Feb 4;20:1737054. doi: 10.3389/fnins.2026.1737054 (PMC12913503; doi:10.3389/fnins.2026.1737054)
Supplement: Supplementary file 1 [file Table_1.docx]

**Table 1.** Non-significant relationships between rCBF in lobes and clinical variables (Bonferroni corrected)

| **Clinical Variables:** | **Frontal Left** *(r, p)* | **Frontal Right** *(r, p)* | **Limbic Left** *(r, p)* | **Limbic Right** *(r, p)* | **Occipital Left** *(r, p)* | **Occipital Right** *(r, p)* | **Parietal Left** *(r, p)* | **Parietal Right** *(r, p)* | **Temporal Left** *(r, p)* | **Temporal Right** (r, p) |
| --- | --- | --- | --- | --- | --- | --- | --- | --- | --- | --- |
| **FRS Percentage** | 0.347, 0.089 | 0.345, 0.091 | 0.137, 0.515 | 0.135, 0.521 | 0.029, 0.890 | 0.118, 0.573 | 0.025, 0.907 | 0.121, 0.565 | 0.068, 0.745 | 0.086, 0.684 |
| **FBI Negative Symptoms** | -0.397, 0.050 | -0.341, 0.095 | -0.022, 0.916 | -0.194, 0.353 | -0.134, 0.524 | -0.176, 0.401 | -0.260, 0.210 | -0.352, 0.084 | -0.196, 0.348 | -0.126, 0.547 |
| **FBI Positive Symptoms** | -0.484, 0.014 | -0.492, 0.012 | -0.420, 0.037 | -0.087, 0.680 | -0.218, 0.296 | -0.233, 0.262 | -0.436, 0.239 | -0.483, 0.095 | -0.237, 0.253 | -0.191, 0.360 |
| **Decline in Conscientiousness** | -0.035, 0.178 | 0.017, 0.290 | -0.134, 0.201 | -0.123, 0.798 | -0.310, 0.731 | -0.006, 0.937 | -0.537, 0.329 | -0.275, 0.565 | -0.304, 0.525 | -0.024, 0.478 |
| **Decline in Extraversion** | -0.172, 0.411 | -0.325, 0.112 | -0.072, 0.731 | -0.122, 0.560 | -0.406, 0.867 | -0.355, 0.987 | -0.311, 0.131 | -0.280, 0.175 | -0.062, 0.768 | -0.036, 0.865 |
| **MoCA** | 0.078, 0.711 | 0.052, 0.806 | 0.011, 0.957 | 0.025, 0.907 | 0.130, 0.536 | 0.130, 0.536 | 0.132, 0.530 | 0.028, 0.894 | 0.187, 0.372 | 0.181, 0.388 |
| **Taylor-Copy Condition** | 0.083, 0.692 | 0.142, 0.499 | 0.220, 0.290 | -0.021, 0.921 | 0.088, 0.677 | 0.054, 0.798 | 0.228, 0.273 | 0.214, 0.304 | 0.166, 0.427 | 0.055, 0.793 |
| **Story Memory** | 0.278, 0.178 | 0.214, 0.303 | 0.359, 0.078 | 0.213, 0.306 | 0.377, 0.864 | 0.228, 0.273 | 0.454, 0.023 | 0.375, 0.065 | 0.416, 0.039 | 0.347, 0.053 |
| **TMT-Part B** | -0.318, 0.122 | -0.265, 0.201 | -0.017, 0.937 | -0.023, 0.912 | -0.303, 0.141 | -0.212, 0.310 | -0.439, 0.028 | -0.362, 0.075 | -0.087, 0.680 | -0.097, 0.644 |
| **R4Alz-R Battery** | -0.474, 0.035 | -0.424, 0.034 | -0.379, 0.062 | -0.320, 0.119 | -0.467, 0.059 | -0.270, 0.193 | -0.466, 0.039 | -0.388, 0.056 | -0.344, 0.093 | -0.215, 0.303 |
| **TASIT-S Part 2** | 0.464, 0.019 | \|  \| \| --- \|   0.192, 0.357 | 0.318, 0.122 | \|  \| \| --- \|   0.167, 0.427 | 0.144, 0.492 | 0.151, 0.471 | 0.317, 0.122 | 0.315, 0.125 | \|  \| \| --- \|   0.238, 0.251 | 0.142, 0.498 |
